# Machine learning-based radiomics strategy for prediction of acquired EGFR T790M mutation following treatment with EGFR-TKI in NSCLC

**DOI:** 10.1038/s41598-023-50984-7

**Published:** 2024-01-03

**Authors:** Jiameng Lu, Xiaoqing Ji, Xinyi Liu, Yunxiu Jiang, Gang Li, Ping Fang, Wei Li, Anli Zuo, Zihan Guo, Shuran Yang, Yanbo Ji, Degan Lu

**Affiliations:** 1 Department of Respiratory, The First Affiliated Hospital of Shandong First Medical University and Shandong Provincial Qianfoshan Hospital, Shandong Institute of Respiratory Diseases, Shandong Institute of Anesthesia and Respiratory Critical Medicine, 16766 Jingshilu, Lixia, Jinan, 250014 Shandong People’s Republic of China; 2 School of Microelectronics, Shandong University, Jinan, 250100 Shandong People’s Republic of China; 3 Department of Nursing, The First Affiliated Hospital of Shandong First Medical University and Shandong Provincial Qianfoshan Hospital, Jinan, 250014 Shandong People’s Republic of China; 4 Graduate School of Shandong First Medical University, Jinan, 250000 Shandong People’s Republic of China; 5 Department of Radiology, The First Affiliated Hospital of Shandong First Medical University and Shandong Provincial Qianfoshan Hospital, Shandong Medicine and Health Key Laboratory of Abdominal Medicine Imaging, Shandong Lung Cancer Institute, Shandong Institute of Neuroimmunology, Jinan, 250000 Shandong China; 6 Department of Blood Transfusion, The First Affiliated Hospital of Shandong First Medical University and Shandong Province Qianfoshan Hospital, Jinan, 250014 Shandong China

**Keywords:** Diseases, Medical research, Cancer

## Abstract

The epidermal growth factor receptor (EGFR) Thr790 Met (T790M) mutation is responsible for approximately half of the acquired resistance to EGFR-tyrosine kinase inhibitor (TKI) in non-small-cell lung cancer (NSCLC) patients. Identifying patients at diagnosis who are likely to develop this mutation after first- or second-generation EGFR-TKI treatment is crucial for better treatment outcomes. This study aims to develop and validate a radiomics-based machine learning (ML) approach to predict the T790M mutation in NSCLC patients at diagnosis. We collected retrospective data from 210 positive EGFR mutation NSCLC patients, extracting 1316 radiomics features from CT images. Using the LASSO algorithm, we selected 10 radiomics features and 2 clinical features most relevant to the mutations. We built models with 7 ML approaches and assessed their performance through the receiver operating characteristic (ROC) curve. The radiomics model and combined model, which integrated radiomics features and relevant clinical factors, achieved an area under the curve (AUC) of 0.80 (95% confidence interval [CI] 0.79–0.81) and 0.86 (0.87–0.88), respectively, in predicting the T790M mutation. Our study presents a convenient and noninvasive radiomics-based ML model for predicting this mutation at the time of diagnosis, aiding in targeted treatment planning for NSCLC patients with EGFR mutations.

## Introduction

Lung cancer is one of the most commonly diagnosed cancers and the leading cause of cancer-related death worldwide^1^. Non-small cell lung cancer (NSCLC) accounts for approximately 85% of primary lung cancer^2^. Although considerable progress has been achieved in the treatment of NSCLC over the past two decades, the overall cure and survival rates for NSCLC remain low, particularly in patients with advanced/metastatic disease^3^.

Epidermal growth factor receptor (EGFR) mutations are identified in approximately 20% of patients with NSCLC in the Caucasian population, and up to 40% in the Asian population^4,5^. First-generation (gefitinib, erlotinib) and second-generation (afatinib, dacomitinib) EGFR-tyrosine kinase inhibitors (TKIs) serve as standard first-line therapy for treatment-naive patients with sensitizing EGFR mutation-positive advanced/metastatic NSCLC patients because these TKIs have provided patients with clinical benefit, such as high response rate and prolonged progression-free survival (PFS) versus platinum-based doublet chemotherapy^6–10^. However, most patients develop resistance within 10–14 months after initial treatment^11^.

The most frequently identified mechanism of acquired TKI resistance is an EGFR Thr790Met (T790M) point mutation within exon 20, which confers drug resistance by increasing ATP affinity^12^. Osimertinib, a third-generation EGFR-TKI which selectively inhibits both EGFR sensitizing mutations and EGFR T790M resistance mutations, had significantly greater efficacy than platinum-based doublet chemotherapy in T790M-positive advanced NSCLC patients who had progressed during first-line EGFR-TKI therapy^13^. At present, osimertinib is a treatment choice for patients with EGFR mutation-positive advanced NSCLC in the first-line setting, and for patients with T790M positive NSCLC following disease progression after first-line EGFR-TKIs^12,14^.

Although 3 generations of EGFR-TKIs are currently available for the treatment of EGFR mutation-positive NSCLC, the optimal sequence of administrating these drugs to maximize the duration of the EGFR signaling inhibition remains still uncertain^15,16^. For patients who are likely to develop the EGFR T790M mutation, a sequencing strategy of the first- or second-generation TKIs followed by osimertinib has shown promising outcomes^16,17^. For T790M-negative patients, osimertinib may be a first-choice TKI^14,18^. Therefore, it is of great importance to identify patients at the time of diagnosis who would be likely to acquire T790M after treatment with a first- or second-generation EGFR-TKI, as this will enable appropriate screening for improved treatment outcomes.

Radiomics is a rapidly evolving field related to the computerized extraction and analysis of data from digital medical images, which offers unique potential to significantly improve the efficiency and accuracy of lung cancer screening, as well as enhance clinical decision-making^19–21^. By extracting imaging information from magnetic resonance imaging (MRI), computed tomography (CT), and positron-emission-tomography (PET), radiomics analysis can be performed to characterize histology and genotype of nodules, identify patient candidate for molecular targeted therapy and immunotherapy, predict treatment response and potential side effects of radiation and immunotherapy, and even differentiate lung injury from recurrence^22–26^. In a previous study, we built a model integrating radiomics and clinical variables for prediction of EGFR mutation and achieved an AUC of 0.86^27^. Yang and colleague collected thoracic CT scans from patients who had confirmed progression on first- or second-generation TKIs and developed a model based on radiomics features and clinical data to detect acquired T790M mutation^28^. They reported an AUC of 0.71 for predicting T790M mutation in patients with advanced lung adenocarcinoma who experienced progression after first- or second-generation EGFR-TKI therapy. However, it is even more important to identify acquired T790M mutation in untreated NSCLC patients in order to optimize the sequence of EGFR-TKI administration. Yet, to date, very few studies have investigated whether radiomics may be capable of predicting the likelihood of developing T790M in treatment-naïve NSCLC patients after a first- or second-generation EGFR-TKI therapy. Therefore, the aim of this study is to establish a radiomics-based model for predicting acquired EGFR T790M mutation in patients with advanced or metastatic NSCLC harbouring an EGFR-activating mutation.

## Material and methods

### Patients

The study population was retrospectively selected from patients with NSCLC at the First Affiliated Hospital of Shandong First Medical University (Jinan, China) between Jan. 2018 and Dec. 2021. This study received approval from the institutional review board of the First Affiliated Hospital of Shandong First Medical University, with a waiver for the requirement of informed consent. Patients who were: (1) histologically diagnosed with primary NSCLC, (2) with known EGFR sensitive mutation, (3) treatment-naïve subjects at the time of diagnosis of NSCLC, (4) classified as unresectable stage III or metastatic (stage IV) according to the Eighth Edition of the Lung Cancer Stage Classification^29^, (5) receiving chest CT scan prior to biopsies, (6) treated with either gefitinib, erlotinib, icotinib, or afatinib as first-line EGFR-TKI therapy, (7) receiving re-biopsy after TKI failure and were tested for EGFR T790M mutation, met the inclusion criteria and were included. The exclusion criteria were given as follows: (1) lack of clinical and demographic data, such as age, gender, smoking status, stage, and levels of serum tumor marker, (2) difficulty in drawing regions of interest (ROIs), (3) poor quality of CT images. In the end, 274 patients were included and randomly assigned to a training cohort (n = 192) and a validation cohort (n = 82). The detailed process of screening and grouping of NSCLC cases is shown in Supplementary Fig. 1.

### Analysis of EGFR Mutation

The tumor specimen was examined at diagnosis for EGFR mutations in exons 18, 19, 20, and 21 by an amplification refractory mutation system (ARMS) real-time technology using Human EGFR Gene Mutations Fluorescence Polymerase Chain Reaction (PCR) Diagnostic Kit (Amoy Diagnostics Co., Ltd, Xiamen, China) or next-generation sequencing (NGS) (Xiansheng Medical Diagnosis Co., Ltd, Nanjing, China). The presence of T790M mutation was detected on relapsed tumor tissue or circulating free DNA (cfDNA) from plasma sample also by ARMS-PCR or NGS, as described elsewhere^30,31^.

### Image acquisition

All CT scans were performed prior to any treatment for lung cancer using two CT scanners (GE Healthcare, Milwaukee, WI, USA; United Imaging, Shanghai, China). The scanning parameters were as follows: the tube voltage, 120 kVp; tube current, 160–300 mA; detector collimation, 64 or 128 × 0.625 mm; field of view, 350 × 350 mm; the pitch, 0.992:1; and matrix of 512 × 512. All images were reconstructed with a section thickness of 2 mm and subsequently stored in DICOM format in the Picture Archiving and Communication Systems (PACS) using mediastinal (width, 360 HU; level, 50 HU) and lung (width, 1500 HU; level, -650 HU) window settings.

### Image preprocessing

Due to the use of different CT scans in the present study, image preprocessing prior to segmentation and feature extraction was performed to make the radiomic features more robust and more suitable for further analysis^32^. A resampling method was used in this process according to the modified protocol reported previously^33^. In brief, the CT image pixel values were first converted from radiodensity to Hounsfield Units (HU) using the metadata attributes of the scans. Then, the entire dataset, including tumor masks, was resampled to standardize image representations. The spacing between slices and pixel spacing were set to 1 mm and [1.0, 1.0] mm, respectively. Each slice dimension was adjusted accordingly to match the new spacing, and the resampled image was obtained through interpolation.

### Tumor segmentation

Tumor regions of interest (ROI) were manually segmented slice by slice by a senior radiologist with over 10-year experience of CT interpretation using ITK-SNAP (Version 3.6, www.itksnap.org)^34,35^. The ROI segmentation was subsequently verified by another chest radiologist with 15-year experience. Both radiologists had no knowledge of the clinical data and mutational status. When one patient has multiple lesions, the radiologist only delineates the tumor area where the biopsy was performed. To assess the consistency of segmentation between the two radiologists, the intra-group correlation coefficient (ICC) for each feature was calculated^36,37^. Only features with an ICC greater than 0.85 were deemed to have high stability and were remained for the further analysis.

### Radiomic feature extraction

In this study, radiomics features were extracted from each three-dimensional (3D) ROI using Pyradiomics library (http://pyradiomics.readthedocs.io/en/latest/index.html), which was in conformance with the Image Biomarker Standardization Initiative 38. These features can be classified as 3 categories: first-order statistics, shape-based, and textural feature^39^. The textural feature category consists of gray level co-occurrence matrix (GLCM), gray level run length matrix (GLRLM), gray level size zone matrix (GLSZM), gray level dependence matrix (GLDM), and neighboring gray tone difference matrix (NGTDM). Moreover, two filters (including wavelet and Laplacian of Gaussian were also utilized to obtain transformed images from the original CT images. By decomposing the image with wavelet transform, high (H) or low (L) pass filter in 3D were used and 8 kinds of combinations were obtained: LHL, HHL, HLL, HHH, HLH, LHH, LLH, and LLL. To emphasize areas of gray level change, the LoG filter was applied to the input image and yield a derived image for each sigma value specified^40^. Five filters with different sigma values (sigma = 1.0 mm, 2.0 mm, 3.0 mm, 4.0 mm, 5.0 mm) were applied in the present study.

### Radiomic feature selection

Although numerous radiomic features were extracted and quantified, not all of them showed an association with the status of T790M mutation. Therefore, feature selection is of great importance to improving the generalization ability and optimizing the model. In this study, the z-score method was initially used to standardize all radiomics features in order to reduce the redundancy between these features. Subsequently, the Wilcoxon rank sum test was used to retain features with a *P*-value < 0.05^41^. Then, the least absolute shrinkage and selection operator (LASSO) algorithm, which was suitable for high-dimensional, small-sample size data with the problem of collinearity^42,43^, was employed to select potential features related to T790M mutational status. LASSO can identify the most predictive features while minimizing overfitting and selection bias. Finally, backward stepwise logistic regression analysis was conducted to select the variables for model building. The termination rule for this process was based on the likelihood ratio test with Akaike’s information criterion (AIC) minimum method^44^.

### Models establishment and performance evaluation

After feature selection, 7 machine learning (ML) methods were imported from the scikit-learn library in Python software to construct models^45^. These algorithms included decision tree (DT), k nearest neighbors (KNN), logistic regression (LR), naïve Bayes (NB), random forest (RF), support vector machines (SVM), and extreme gradient boosting (XGBoost, XGB). The performances of these models were first assessed by analysis of area under the curve (AUC) of receiver operating characteristic (ROC) curve, sensitivity, and specificity in the validation cohort. Fivefold cross-validation was simultaneously applied to evaluate all results. Then, the optimal model was selected for further analysis.

Several clinical features are associated with mutant EGFR status in NSCLC patients^46,47^. Therefore, some clinical factors were included in the analysis. They consisted of age, gender, smoking status, performance status (at biopsy), stage of disease, serum level of tumor markers, and the initial response to first- or second-generation EGFR-TKI. The Chi-square and Student’s t-tests were first used to screen clinical factors related to EGFR T790M mutation and those with a *P*-value lower than 0.05 were retained for further analysis.

To enhance the prediction accuracy even further, the optimal classifier and significant clinical features correlated to EGFR T790M mutation were integrated to establish the combined models. The predictive performances of these models were also evaluated based on the AUC of ROC curve analysis.

### Nomogram construction

**Figure 1:**
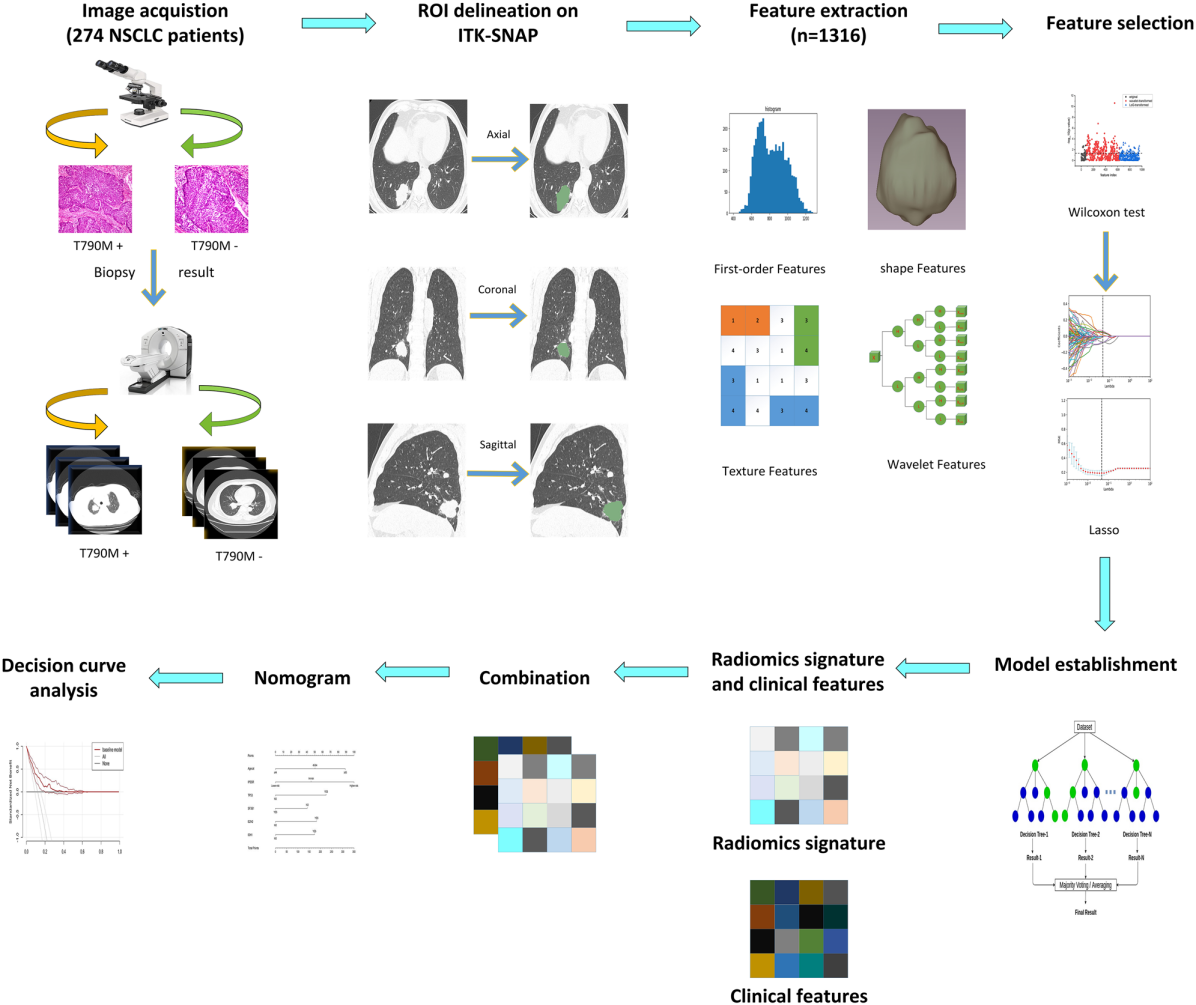
Workflow of the radiomic analysis.

Nomograms utilize multiple prognostic and determinant variables to establish a statistical prognostic model which can generate an individual probability of a clinical event, thus aiding clinical decision making^48^. In the present study, a nomogram model was developed based on a multivariable logistic analysis. For each patient, a radiomics score (Rad-score) was calculated by assigning weights to discriminating radiomic features based on their respective coefficients. The radiomics signature and clinical factors were incorporated into a nomogram model designed to predict EGFR T790M mutation in the training cohort. The predictive accuracy of the model was evaluated by a calibration curve. Decision curve analysis (DCA) was conducted to examine the performance of a model to predict EGFR T790M mutation by quantifying the net benefits of 2 models in both the training and validation cohorts^33^. The workflow of the radiomic analysis is depicted in Fig. 1.

### Statistical analysis

Statistical analysis was conducted using PRISM version 6 (GraphPad, La Jolla, CA, USA). Quantitative data were compared using Student’s t-test, and categorical data were compared using the χ^2^ test to identify any baseline differences. The discrimination performance of models was assessed by the ROC curve and the AUC, sensitivity, and specificity of the model were calculated. All statistical tests were two-tailed, and statistical significance was set a priori at 0.05.

### Ethics statement

The study was reviewed and approved by the Institutional Review Committee of the First Affiliated Hospital of Shandong First Medical University (Jinan, China) (approval no. 2019-S-306). Written informed consent for participation is not required for this study in accordance with national legislative and institutional requirements.

## Results

### Clinical characteristics of patients

The baseline clinical characteristics of the enrolled patients were shown in Table 1. No evident differences were found with respect to the age, smoking status , performance status (at biopsy), stage of disease, pathological type, EGFR mutation status, and serum level of CEA, NSE, CYFRA 21-1, and Pro-GRP between patients with the EGFR T790M mutation and those without (*P* > 0.05). However, a substantial difference was observed in the initial response to first- or second-generation EGFR-TKI between T790M-positive and T790M-negative patients. In both the training cohort and the validation cohort, the objective remission rate (ORR) in the T790M-positive group was significantly higher than that in the T790M-negative group (*P* < 0.05). In addition, a higher proportion of male individuals were observed in T790M-positive patients compared to T790M-negative patients in both the training cohort and the validation cohort (*P* < 0.05).

**Table 1:** Clinicopathological characteristics of patients enrolled in this study.

Variable	Training cohort		*p*	Validation cohort		*p*	*p*
	T790M-positive (N = 90)	T790M-negative (N = 102)		T790M-positive (N = 38)	T790M-negative (N = 44)		
Age (y, mean ± SD)	65.30 ± 9.90	64.16 ± 10.72	0.45	65.26 ± 10.81	66.11 ± 10.98	0.73	0.46
Sex, *n* (%)			0.04			0.03	0.01
Male	52 (57.78)	43 (42.16)		26 (68.42)	19 (43.18)		
Female	38 (42.22)	59 (57.84)		12 (31.58)	25 (56.82)		
Smoking Status, *n* (%)			0.66			0.82	0.06
Smoker	47 (52.22)	49 (48.04)		20 (52.63)	21 (47.73)		
Never smoker	43 (47.78)	53 (51.96)		18 (47.37)	23 (52.27)		
Performance status (at biopsy)			0.55			0.65	0.05
0–1	57 (63.33)	69 (67.65)		24 (63.16)	30 (68.18)		
≥ 2	33 (36.67)	33 (32.35)		14 (36.84)	14 (31.82)		
Pathological type			0.69			0.64	0.94
Adenocarcinoma	85 (94.45)	96 (94.12)		36 (94.74)	41 (93.18)		
Adenosquamous carcinoma	3 (3.33)	5 (4.90)		2 (5.26)	2 (4.55)		
Squamous carcinoma	2 (2.22)	1 (0.98)		0 (0.00)	1 (2.27)		
Stage, *n* (%)			0.72			0.79	0.87
III	20 (22.22)	20 (19.61)		8 (21.05)	8 (18.18)		
IV	70 (77.78)	82 (80.39)		30 (76.95)	36 (81.82)		
EGFR mutation			0.30			0.50	0.89
Exon 19 deletion	53 (58.89)	68 (66.67)		22 (57.89)	29 (65.91)		
Exon 21 L858R	37 (41.11)	34 (33.33)		16 (42.11)	15 (34.09)		
Serum level of tumor marker (mean ± SD)							
CEA	68.33 ± 85.64	59.56 ± 84.91	0.48	43.91 ± 42.51	48.66 ± 63.23	0.69	0.09
NSE	18.08 ± 19.38	15.11 ± 6.20	0.14	20.33 ± 23.13	21.62 ± 43.28	0.87	0.13
CYFRA 21–1	5.35 ± 6.69	3.25 ± 3.43	0.01	4.19 ± 3.90	5.20 ± 5.78	0.36	0.47
SCC	0.93 ± 0.88	0.96 ± 1.02	0.82	0.73 ± 0.42	0.72 ± 0.47	0.93	0.04
Pro-GRP	47.68 ± 51.69	42.69 ± 44.04	0.47	51.15 ± 68.95	38.81 ± 13.09	0.23	0.90
Initial EGFR-TKI response			0.02			0.01	0.04
CR or PR	65 (72.22)	57 (55.88)		33 (86.84)	26 (59.09)		
SD or PD	25 (27.78)	45 (44.12)		5 (13.16)	18 (40.91)		

*EGFR* epidermal growth factor receptor, *CEA* carcinoembryonic antigen, *NSE* neuron-specific enolase, *CYFRA 21–1* fragment of cytokeratin subunit 19, *SCC* squamous cell carcinoma antigen, *Pro-GRP* pro-gastrin-releasing peptide, *CR* complete response, *PR* partial response, *SD* stable disease, *PD* progressive disease.

### Feature extraction and selection

In total, 1316 radiomic features were successfully extracted from each patient’s ROI. Clinical features included gender and initial response to first- or second-generation EGFR-TKI. The ICC (= mean ± SD) for radiomics features in each group were calculated. Shape-based features (ICC = 0.92 ± 0.07), first-order statistics (ICC = 0.93 ± 0.03), GLRLM-derived texture features (ICC = 0.96 ± 0.04), GLCM-derived texture features (ICC = 0.97 ± 0.02), met the criteria for high stability (ICC > 0.85), while GLSZM-derived texture features (ICC = 0.56 ± 0.44), GLDM-derived texture features (ICC = 0.36 ± 0.44), NGTDM-derived texture features (ICC = 0.59 ± 0.32), LoG features (ICC = 0.77 ± 0.35), and wavelet (ICC = 0.78 ± 0.36) features did not. Out of the initial 1316 features, 955 (72.60%) were identified as stable and retained. These features comprised 13 shape-based features, 18 first-order features, 16 GLRLM features, 24 GLCM features, 6 GLSZM features, 5 GLDM features, 3 NGTDM features, 544 wavelet transformed features, and 326 LoG transformed features. The histogram of the ICC values of the radiomic features was shown in Supplementary Fig. 2.

**Figure 2:**
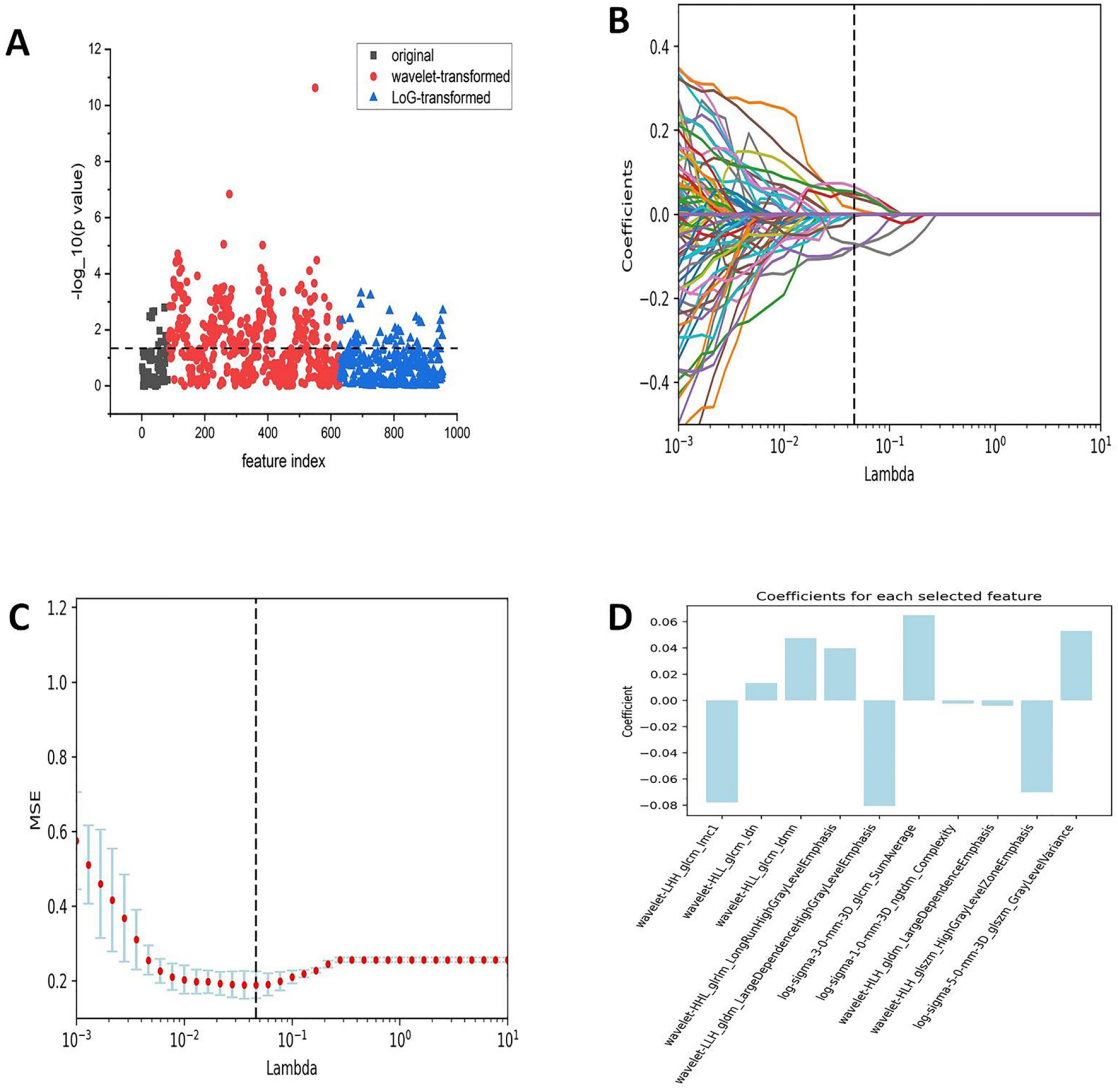
Radiomics feature selection process. (**A**) The features were screened using the Wilcoxon rank-sum test, and the test level was 0.05. (**B**, **C**) The LASSO was used to further filter the most relevant features. (**D**) Coefficients for each selected radiomics feature. MSE: mean square error, LASSO: least absolute shrinkage and selection operator.

**Figure 3:**
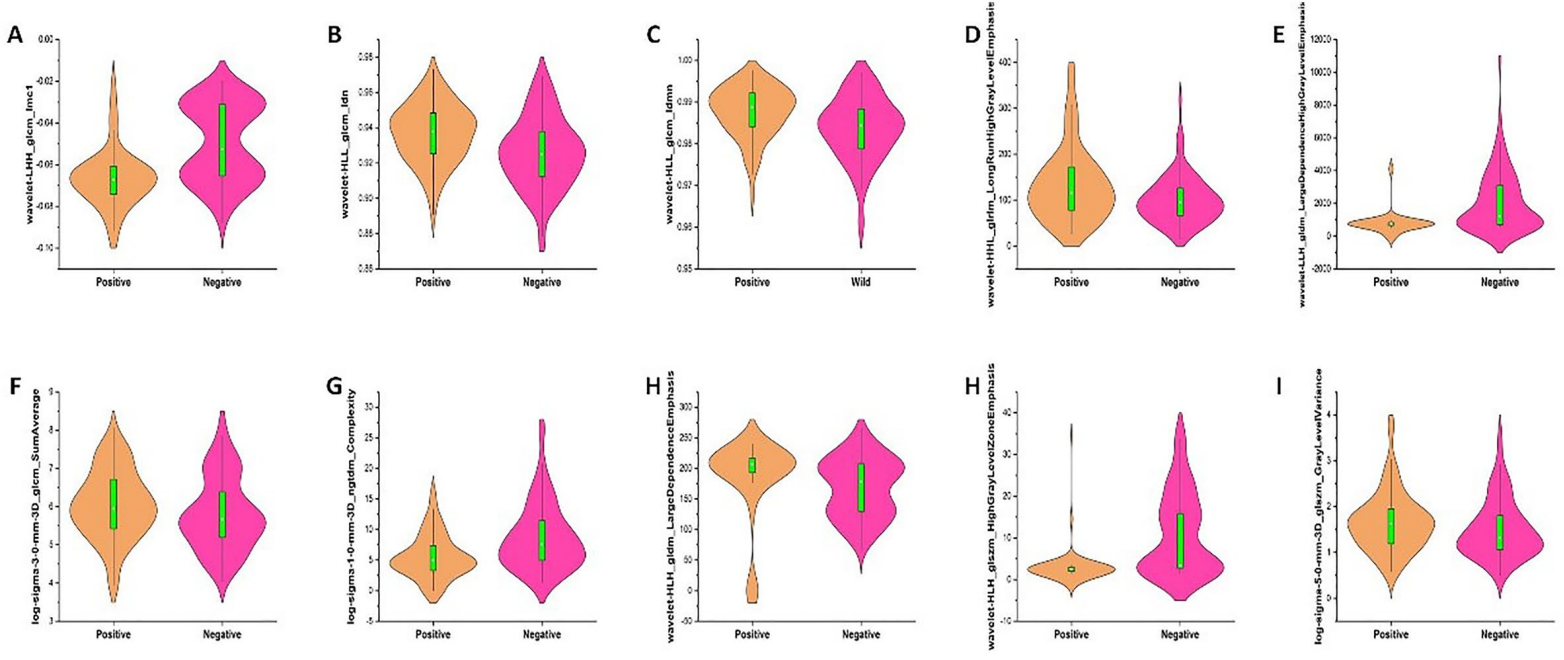
Boxplot showing 10 radiomics features with significant difference between the T790M-positive and T790M-negative in the training set.

Subsequently, the dimensionality reduction was performed and the coefficient for each selected feature was illustrated in Fig. 2. As shown in Fig. 2B,C, when the variable is equal to 10, the error classification value is lower. Thus, the 10 features were selected to build the LASSO logistic regression model. The coefficients for each selected radiomics feature are shown in Fig. 2D. Figure 3 shows the 10 radiomics features were significantly different between the T790M-positive and T790M-negative patients in the training set.

### Predictive performance assessment of models

**Figure 4:**
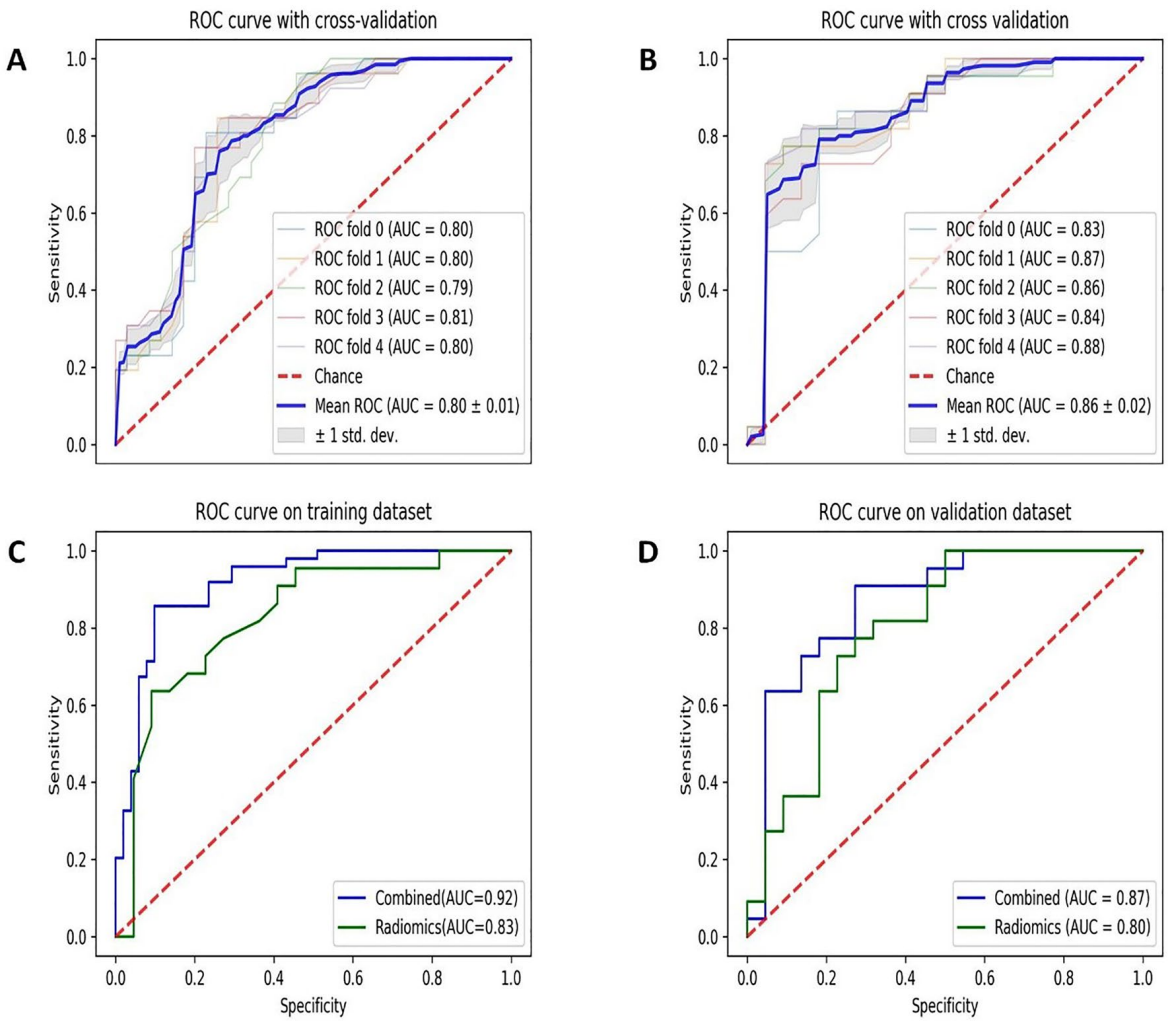
ROC curves of models. (**A**) The fivefold cross-validated ROC curve of model RF. (**B**) The fivefold cross-validated ROC curve of combined model. (**C**) ROC curve of RF and combined model on the training dataset. (**D**) ROC curve of RF and combined model on the validation dataset.

The predictive performance of all 7 models based on radiomics was presented in Supplementary Fig. 3. Among these models, the ML method of RF exhibited superior performance compared to the others in the validation cohorts. The parameters of the RF model were set as follows: the number of trees was 2, and the maximum depth was 4 (Supplementary Fig. 4). In terms of predicting EGFR T790M mutation, the RF and combined models obtained an AUC, sensitivity, specificity, and accuracy of 0.80 (95% confidence interval [CI] 0.79–0.81), 0.86 (0.84–0.89); 0.85 (0.81–0.89), 0.78 (0.72–0.84); 0.70 (0.65–0.74), 0.76 (0.67–0.85); and 0.75 (0.71–0.78), 0.77 (0.73–0.82); respectively using fivefold cross-validation (Fig. 4A,B). Moreover, the combined model, which integrated the radiomics signature derived from RF model and the clinical factors, demonstrated even higher AUC values in the training and validation cohorts: 0.92 and 0.87, respectively Fig. 4C,D.

### Analysis of the radiomic nomogram

**Figure 5:**
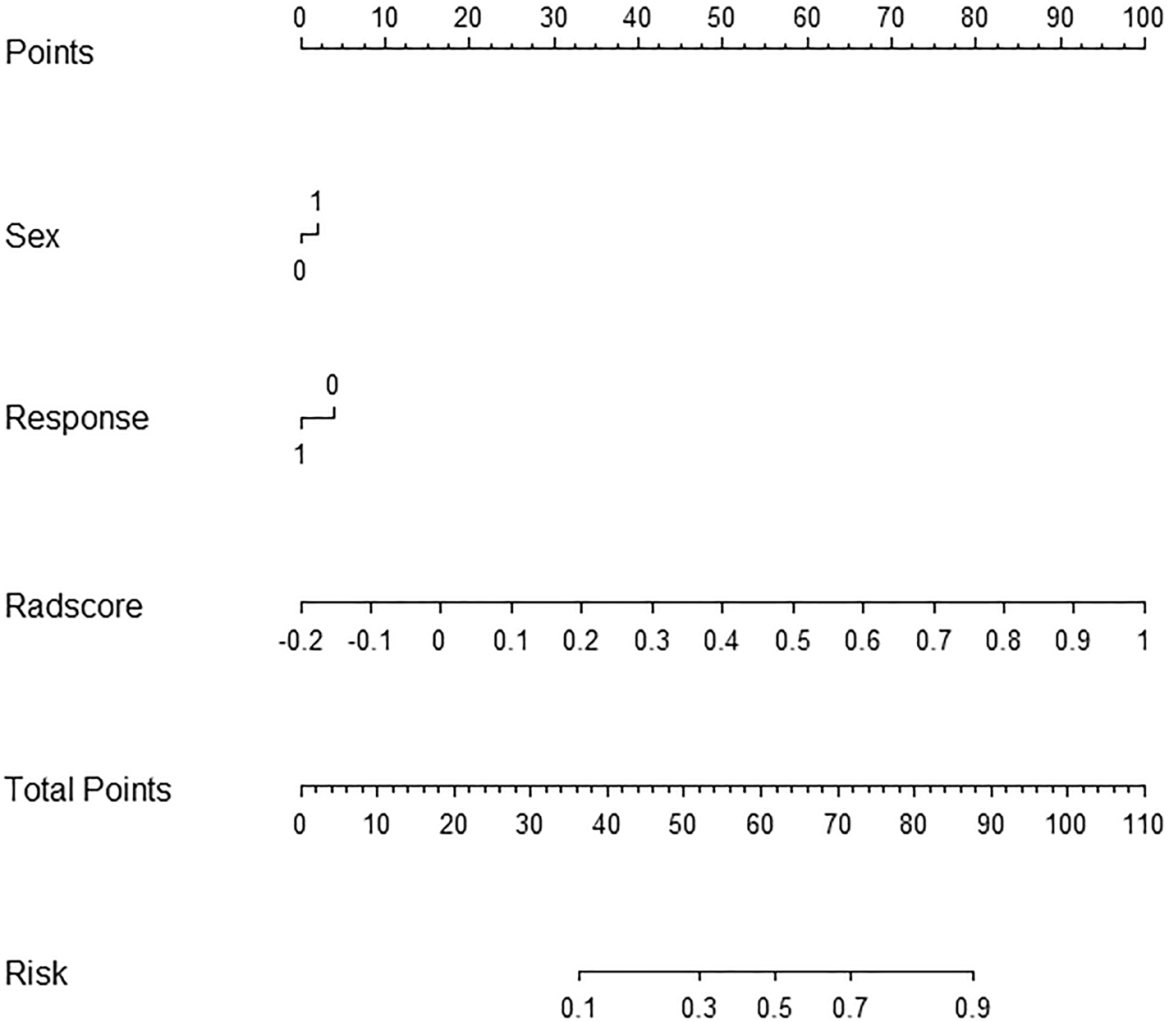
Radiomic nomogram. In the training cohort, the nomogram incorporated the radiomic signature, sex, and the initial response to first- or second-generation EGFR-TKI.

**Figure 6:**
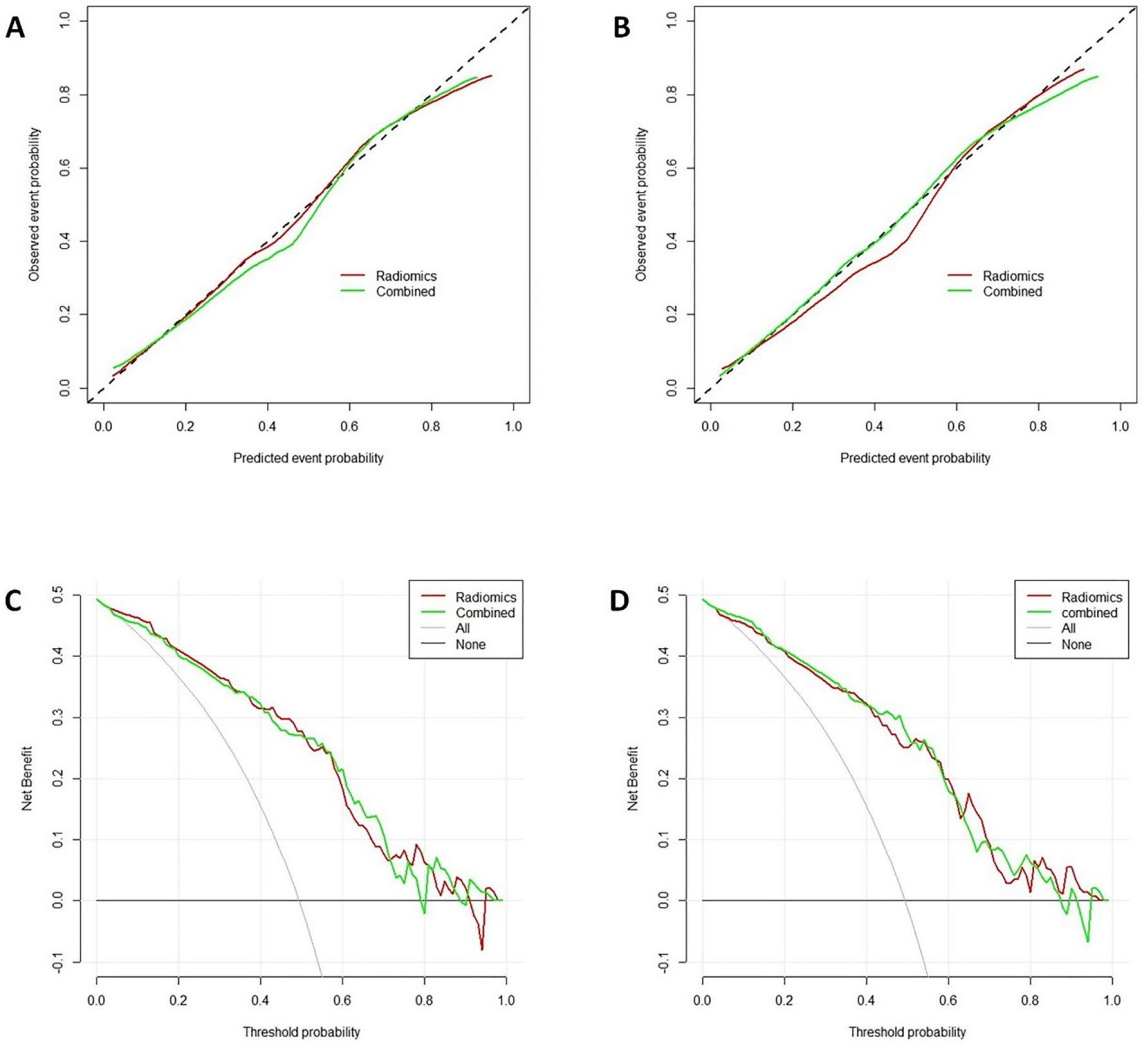
Calibration curve and decision curve analysis (DCA). (**A**) Calibration curve of the nomogram in the training cohort. (**B**). Calibration curve of the nomogram in the validation cohort. (**C**) DCA in the training cohort. (**D**) DCA in the validation cohort.

The radiomic nomogram model developed by integrating the radiomics signatures derived from the RF classifier and clinical predictors achieved higher AUC values in the validation cohort [0.86 (95% CI 0.85–0.88)] (Fig. 5). These findings indicated that the combined model exhibited enhanced predictive capabilities for predicting EGFR T790M mutation. To assess the predictive ability of the model, a calibration curve was plotted. The DCA for the radiomics nomogram and is presented in Fig. 6. Remarkably, the combined model outperformed the other models, as evidenced by its larger area under the decision curve, signifying its superior clinical utility.

## Discussion

In our current study, we built a ML-derived radiomics model utilizing non-invasive CT images to predict EGFR T790M mutation in treatment-naïve patients with NSCLC. This model achieved promising performance in the validation cohort (AUC 0.80, 95% CI 0.79–0.81). In particular, the sensitivity and specificity to identify T790M mutation was 0.85 (0.81–0.89), and 0.70 (0.65–0.74), respectively, indicating a low false-positive and false-negative rate, which would be of help in accurately screening patients with EGFR T790M mutation. Moreover, we proposed a predictive model integrating CT images and clinical features, which achieved an AUC of 0.86 (95% CI 0.81–0.89), a sensitivity of 0.78 (0.72–0.84), and a specificity of 0.76 (0.67–0.85). This model further demonstrated the ability to identify acquired EGFR T790M mutation in patients who received first- or second-generation EGFR-TKIs. Our study unveils the potential of a noninvasive approach in identifying patients who are likely to develop the T790M mutation following treatment with first- and second-generation EGFR-TKIs. Importantly, this is the first study, to the best of our knowledge, investigating the ability of radiomics-based models to predict acquired EGFR T790M mutation in Chinese patients with advanced NSCLC bearing an EGFR-activating mutation by utilizing CT imaging from treatment-naïve patients with NSCLC. Our findings support the feasibility of using this model to identify EGFR T790M mutation, offering valuable guidance in selecting appropriate patients for improved treatment outcomes. This study represents a significant contribution to the field of radiomics research, providing novel insights and potential advancements.

Radiomics is a newly emerging and rapidly progressing field that integrates radiology, oncology, and ML techniques^49^. By utilizing radiomics data, descriptive and predictive models can be developed, providing invaluable diagnostic, prognostic, or predictive information^50^. Moreover, certain radiomics features are even able to detect genomic alterations within tumor DNA, leading to the emergence of ‘radiogenomics’^20^. Although some limitations of the radiogenomic approach may exist, radiogenomics is playing an increasingly important role in precision diagnostics and optimal therapy in lung cancer. For example, radiogenomics can be of help to treatment option and prognosis assessment in NSCLC patients^51,52^. In addition, radiogenomics can aid in evaluating efficacy of therapy and predicting outcomes of patients^51,52^. Therefore, radiogenomics holds great promise for improving decision making, facilitating more precise personalized care, and ultimately improving patient outcomes.

The findings of this study concur with a previous study that highlighted the correlation between radiomics features from baseline chest CT and the subsequent development of the T790M in Caucasian patients with NSCLC after treatment with an EGFR inhibitor^24^. Kim et al. also reported that smaller tumor size and selection of metastatic lung lesions as biopsy targets were associated with the detection of the T790M mutation at re-biopsy for mutational analysis^53^. Furthermore, significant differences in CT imaging were observed between acquired and primary T790M mutations^54^. Our results, combined with previous research, clearly demonstrate the feasibility of identifying individuals who are likely to acquire the T790M mutation after receiving first- or second-generation EGFR-TKIs using baseline CT scans. Intriguingly, Koo et al.^55^ retrospectively analyzed CT findings of NSCLC patients at the initial diagnosis and those at re-biopsy and found that peripheral tumor location with vascular convergence, the presence of a pleural tag, and air bronchogram at the time of re-biopsy were associated with acquired T790M mutation. Yoshida and colleagues^56^ utilized PET scans and found that patients with T790M mutation exhibited lower levels of 18F-2-fluoro-2-deoxyglucose uptake in comparison with those without T790M mutation. These findings further validate the link between radiomics features and development of T790M during treatment with EGFR-TKI. Furthermore, models that integrate both radiomic features and clinical factors demonstrated excellent performance in evaluating the prognosis of metastatic NSCLC patients with *EGFR*-T790M mutation receiving osimertinib treatment^57^.

When some clinical characteristics added to the radiomics based model, the ability of the combined model to predict the T790M mutation has also been assessed in the present study. The combined model, composed of clinicopathologic and CT-radiomic signatures, achieved good detection performance with an AUC 0.87 in the validation datasets. These findings suggests that certain clinical features may contribute to the discrimination of the T790M mutation within the combined model. We identified gender and initial response to first- or second-generation EGFR-TKI as the most influential clinical predictor. These results are consistent with previous studies that reported that gender (male), initial EGFR-TKI response (complete or partial response), progression pattern (solitary lesion progression), longer duration of EGFR-TKI, postsurgery recurrence, may represent useful predictive markers for T790M detection^58,59^. Additionally, Dal Maso et al. demonstrated a correlation between age, type of EGFR mutation at diagnosis, response to first-line treatment, andT790M status^60^. A study by Hou et al. compared the clinical and CT imaging characteristics between primary and acquired EGFR T790M mutations in treatment-naïve patients with NSCLC. They found that patients with primary T790M mutation exhibited earlier tumor stage, higher differentiation, and proportion of lepidic subtype adenocarcinoma in comparison to those with acquired T790M mutations^54^.

Liquid biopsy, which involves analyzing circulating tumor-derived elements in various body fluids, presents a valid alternative to tissue re-biopsy. The liquid biomarkers consisted of circulating free DNA (cfDNA), circulating tumor cells (CTCS), exosomes, and tumor-educated platelets^61^. These components can be easily isolated from almost a wide range of body fluids, including blood, urine, pleural effusion, and ascites^62^. Liquid biopsy offers convenient, non-invasive, and the ability to be performed at multiple time-points. Furthermore, it enables the identification of dynamic changes in gene expression within the tumor and the capturing intrinsic tumor heterogeneity^63^. As a result, liquid biopsy plays an increasingly important role in establishing a diagnosis, detecting molecular characterization, and monitoring mechanisms of resistance in patients with lung cancer^64–66^. The specificity of liquid biopsy for detecting T790M mutation during treatment with first- and second-generation TKIs has also been confirmed^65^. However, there are cases where the results from tissue and cfDNA genotyping do not match, probably due to technological differences or sampling different tumor cell populations^67–69^. Therefore, patients with T790M-negative plasma results still need a tumor biopsy to identify T790M mutation. Consequently, tissue biopsy and blood-based analyses may have complementary roles in evaluating the genetic alterations for these patients^60,68^. In this context, radiomics may offer additional information to assist in the implementation of optimal treatment strategies. Cucchiara et al. developed a mode that integrates liquid biopsy and radiomics, demonstrating good performance in identifying the presence of T790M^70^.

ML is a subset of artificial intelligence (AI) that involves the development of algorithmic models to identify patterns and relationships in data^71^. The main aim of ML techniques is to create models that can be applied to perform tasks, such as classification, prediction, or estimation. Our retrospective study revealed the feasibility of using 7 ML approaches to predict EGFR T790M mutations after treatment with a first- or second-generation EGFR-TKI. Among these classifiers, RF classification method exhibited the highest performance, with an AUC of 0.87. This finding accords with a study by Saini R et al.^72^, which reported that RF-based radiomics classifier achieved the best performance (AUC = 0.776) in predicting the Ki-67 expression level in NSCLC. Parmar et al.^73^ also found that RF classification method showed highest prognostic performance in predicting 2-year patient survival in patients with NSCLC. In addition, RF-based models had best performance in identifying new potent EGFR inhibitors against the resistant T790M mutant^74^. RF is considered a favorable ML method due to its simple structure, ability to deal with both regression and classification issues, and higher efficiency than compared to methods^75^. In our study, the RF-based model which integrated the radiomics signatures and the clinical factors achieved an AUC of 0.87. In terms of interpretability, the predictions made by the RF model can be meaningfully explained from a biological standpoint as well^76^. Additionally, the robustness of the features used in the current study was ensured through the utilization of five-fold cross-validation.

Several limitations of this study should be noted. Firstly, the retrospective nature of the analysis may inevitably result in patient selection bias. Another limitation is the relatively small sample size of patients harboring T790M mutation, which may limit the power of these analyses. However, although larger data sets are more likely to have higher statistical power, radiomics analyses can still be performed with as few as 100 patients^77^. Further studies with large sample size should be conducted to assess the clinical applications of our models. Thirdly, because all of subjects who were involved in this study were Chinese, generalization of these results should not be made beyond this population. Further studies are required to verify these findings within other racial and ethnic population. Finally, manual segmentation of ROI is both tedious and time consuming, and there may be significant variability among different observers. However, manual segmentation can still be a simple and reliable method, and its reproducibility can be evaluated by interobserver reproducibility analysis^78^.

In conclusion, this study demonstrates the feasibility of non-invasively predicting EGFR T790M mutation at diagnosis in NSCLC patients following treatment with a first- or second-generation EGFR-TKI using a ML model integrating radiomic features and clinical characteristics. Our results are promising and warrant validation in a larger sample size. These findings indicate that utilizing this method to detect EGFR-T790M mutation could potentially facilitate the selection of accurate and personalized treatment strategies for patients with NSCLC.

### Supplementary Information

Supplementary Figure S1.

Supplementary Figure S2.

Supplementary Figure S3.

Supplementary Figure S4.

Supplementary Legends.

## Data Availability

The original data supporting the conclusions of this paper will be provided unreservedly by the authors to any qualified researcher. The datasets used and/or analyzed during the current study are available from the corresponding author.
