# Supplementary figures and images for: Machine learning-based radiomics strategy for prediction of acquired EGFR T790M mutation following treatment with EGFR-TKI in NSCLC
Source: Sci Rep. 2024 Jan 3;14:446. doi: 10.1038/s41598-023-50984-7 (PMC10764785; doi:10.1038/s41598-023-50984-7)

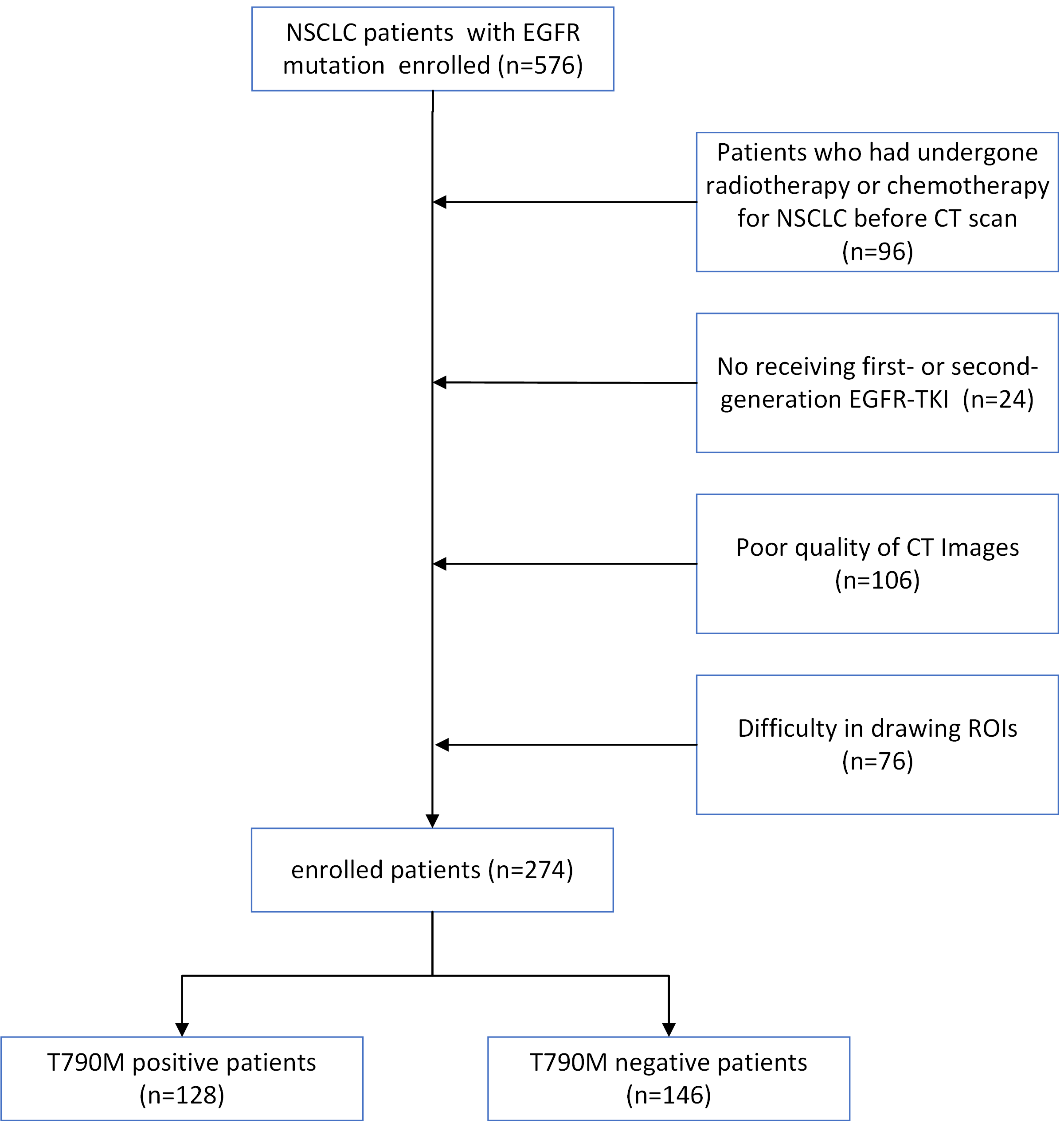

Supplement: Supplementary file 1 — Supplementary Figure S1. [file 41598_2023_50984_MOESM1_ESM.tif]

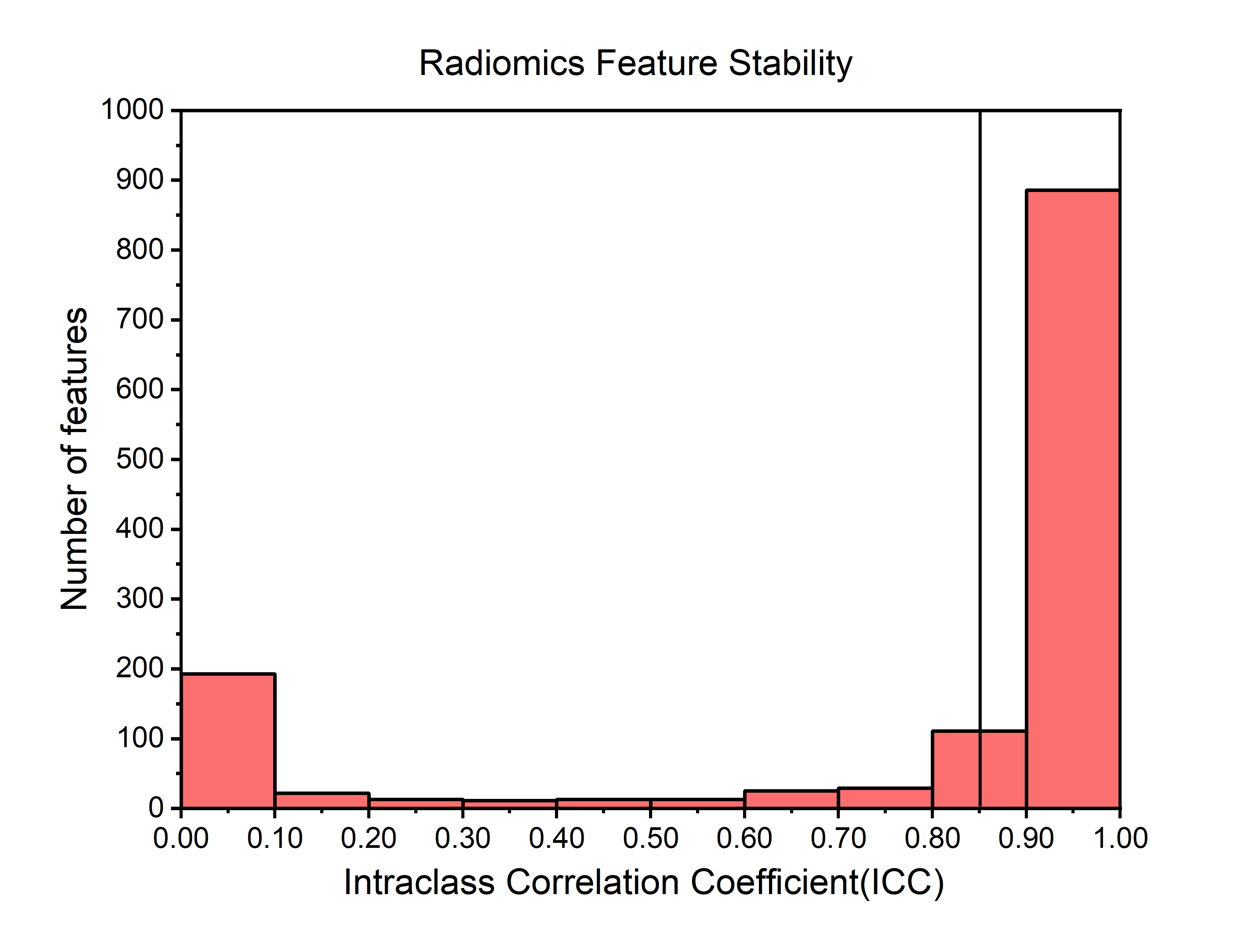

Supplement: Supplementary file 2 — Supplementary Figure S2. [file 41598_2023_50984_MOESM2_ESM.tif]

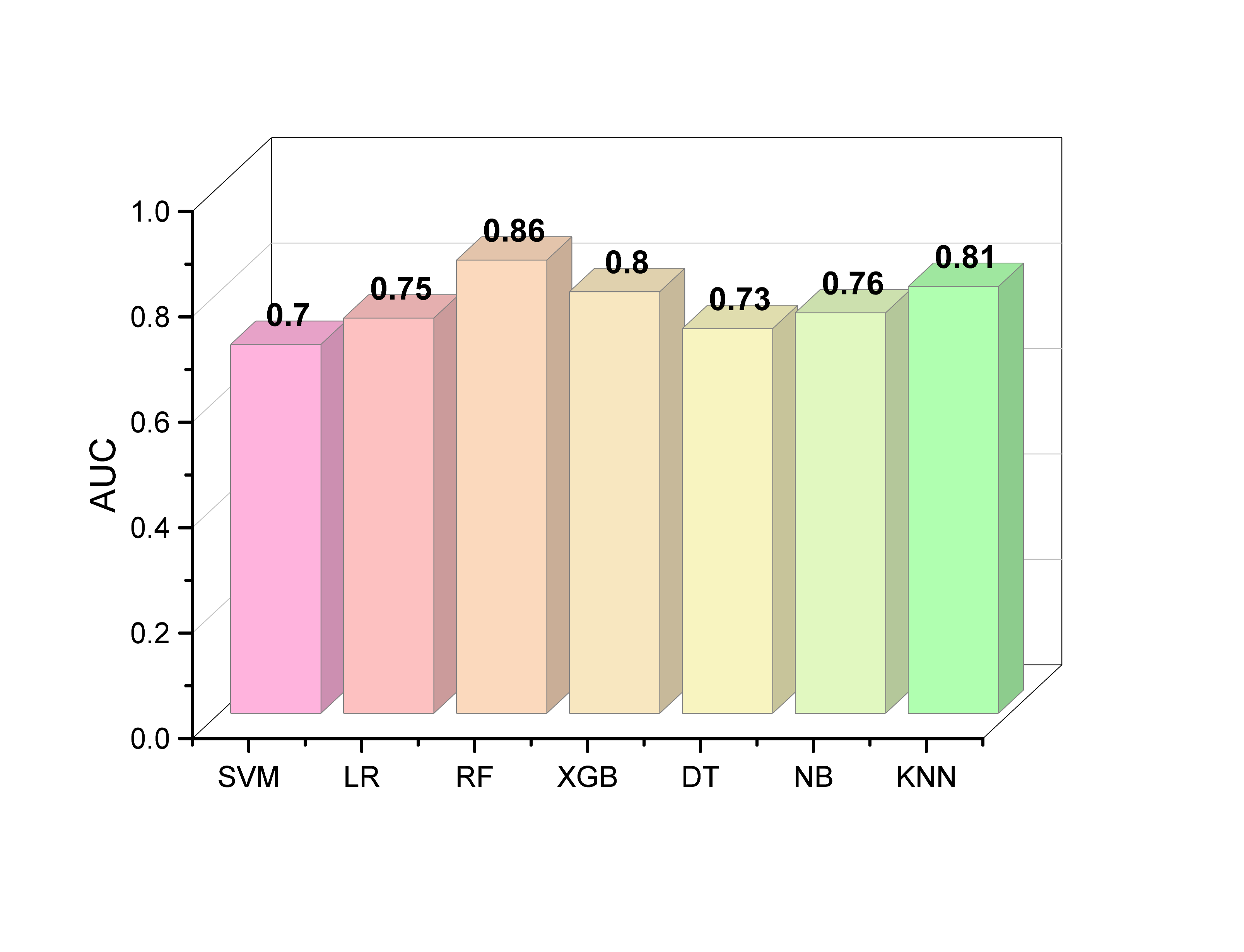

Supplement: Supplementary file 3 — Supplementary Figure S3. [file 41598_2023_50984_MOESM3_ESM.tif]
