# Supplementary material for: Machine learning-based radiomics strategy for prediction of acquired EGFR T790M mutation following treatment with EGFR-TKI in NSCLC
Source: Sci Rep. 2024 Jan 3;14:446. doi: 10.1038/s41598-023-50984-7 (PMC10764785; doi:10.1038/s41598-023-50984-7)
Supplement: Supplementary file 5 — Supplementary Legends. [file 41598_2023_50984_MOESM5_ESM.docx]

Figure S1: Flowchart of NSCLC patient selection.

Figure S2: Histogram of the ICC for radiomics features. ICC: intragroup correlation coefficient.

Figure. S3. The predictive performance of all machine learning methods based on radiomics features.

Figure. S4. The structure of the trees for RF classifier. RF: random forest.
